# Supplementary material for: Rational design of flavivirus E protein vaccine optimizes immunogenicity and mitigates antibody dependent enhancement risk
Source: Nat Commun. 2025 Dec 22;16:11558. doi: 10.1038/s41467-025-67447-4 (PMC12748774; doi:10.1038/s41467-025-67447-4)

SUPPLEMENTARY MATERIAL

Fig. S1. Characterization of ZIKV sE proteins

Fig. S2. Cryo-EM data of ZIKV CC_FLE sE: SMZAb2 complex

Fig. S3. Cryo-EM structural data details of unliganded JEV and WNV CC-FLE sE dimer, related to Fig. 3

Fig. S4. Self-assembly of virus-mimicking supramolecular complexes of CC_FLE sE-PCEP-R848

Fig. S5. Antigenicity validation of the CC_FLE sE-PCEP-R848 complex

Fig. S6. Protection efficacy of passive transferred immune sera

Fig. S7. Dimeric ZIKV CC_FLE sE elicits neutralizing antibody response to epitopes distinctive from monomeric WT sE

Fig. S8. Pregnant mice/fetus protection efficacy

Fig. S9. Immunogenicity of ZIKV CC_FLE sE in OmniMouse animals expressing naïve human immunoglobulin loci

Fig. S10. Single-cell sorting of antigen-specific B cells from splenocytes of immunized OmniMouse OM5.1

Fig. S11. Cryo-EM data of OZ-D4 mAb in complex with stabilized ZIKV E dimer CC_Core

Table S1. Cryo-EM data collection, refinement, and validation statistics for ZIKV, JEV, and WNV CC_FLE sE dimers

Table S2. Comparison of structural parameters of ZIKV and JEV sE dimers in this study and previously published work

Table S3. Genetic and functional characteristics of OmniMouse-derived monoclonal antibodies

Table S4. Cryo-EM data collection, refinement, and validation statistics for the ZIKV CC_Core sE: OZ-D4 Fab complex

Table S5. Monoclonal antibodies used in this study

Table S6. Potential hydrogen bonds and electrostatic interactions of three antibodies SMZAb2, OZ-D4 and EDE1-C8 complexed with ZIKV sE dimers

Uncropped gel image

Fig. S1

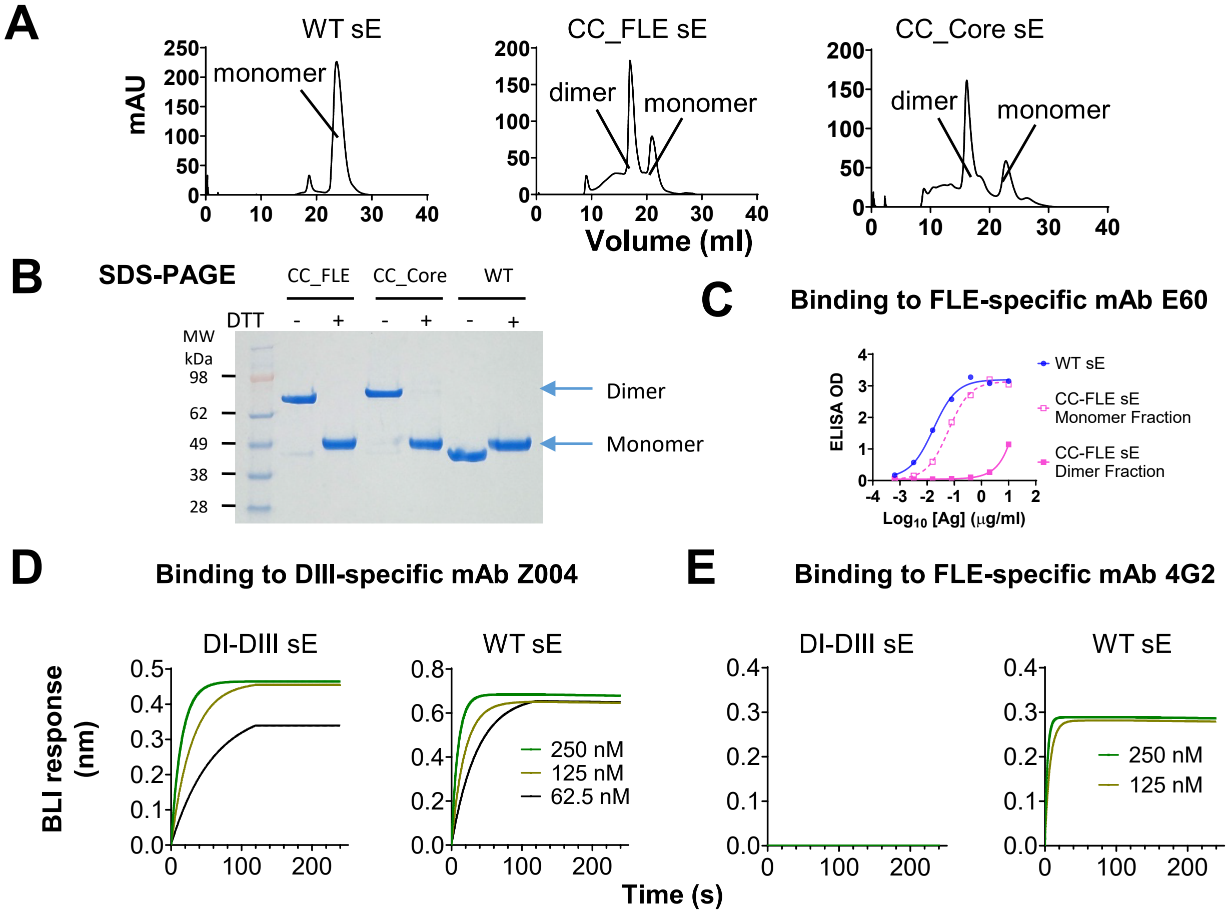

F

mAbs	DI-DIII sE			WT sE		
	K_D (M)	k_{on} (1/Ms)	k_{off} (1/s)	K_D (M)	k_{on} (1/Ms)	k_{off} (1/s)
Z004	<1.0E-12	2.70E+05	<1.0E-07	1.58E-10	4.36E+05	6.88E-05
4G2	no binding			5.79E-11	1.15E+06	6.65E-05

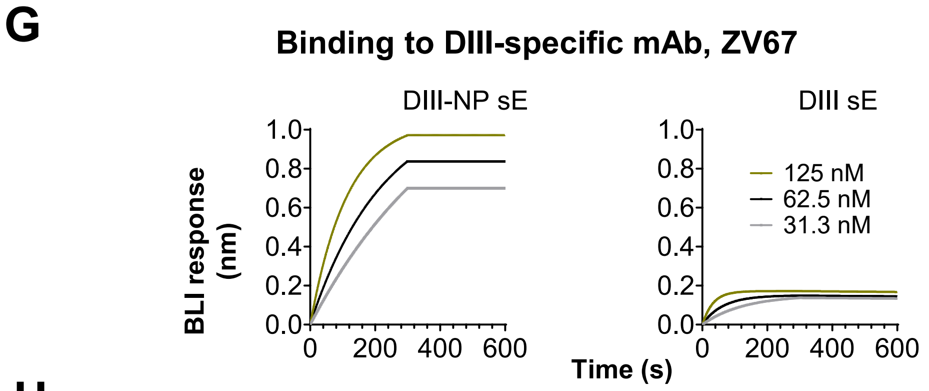

H

mAbs	DIII-NP sE			DIII sE		
	K_D (M)	k_{on} (1/Ms)	k_{off} (1/s)	K_D (M)	k_{on} (1/Ms)	k_{off} (1/s)
ZV67	<1.0E-12	7.09E+04	<1.0E-07	3.45E-10	2.50E+05	8.65E-05

Fig. S1. Characterization of ZIKV sE proteins. (**A**) Size exclusion chromatography (SEC) profiles of sE proteins before SEC purification. (**B**) The dimeric configuration of SEC-purified CC_FLE sE or CC_Core sE is confirmed by SDS-PAGE in the absence of the reducing reagent DTT. In contrast, purified WT sE consistently migrates as a monomer, with an approximate molecular weight of 49 kDa, regardless of the presence or absence of DTT. (**C**) The CC_FLE sE dimer fraction shows poor binding to the FLE-specific mAb E60, whereas the monomer fraction exhibits binding affinity comparable to monomeric WT sE. This suggests that G5C/G102C mutations enhance dimer propensity thereby reduce exposure of the FLE E60 epitope, while the G5C/G102C point mutations in the monomeric context have no effect on E60 binding. (**D, E, G**) Bio-Layer Interferometry (BLI) binding curves for DI–DIII, DIII, and WT sE proteins. Antibodies were captured on anti-human Fc BLI probes and then immersed in wells containing sE at concentrations of 31.25 nM (grey), 62.5 nM (black), 125 nM (olive), and 250 nM (green). (**D**) BLI curves generated with the DIII-specific mAb Z004. (**E**) BLI curves generated with the FLE-specific mAb 4G2. (**F)** Binding kinetic parameters for DI–DIII and WT sEs derived from (D) and (E). (**G**) BLI analysis of DIII-NP and DIII sEs with the DIII-specific mAb ZV67. (**H**) Binding kinetic parameters derived from (G). Each assay was repeated at least twice. Source data are provided as a Source Data file. Related to **Fig. 2**.

Fig. S2.

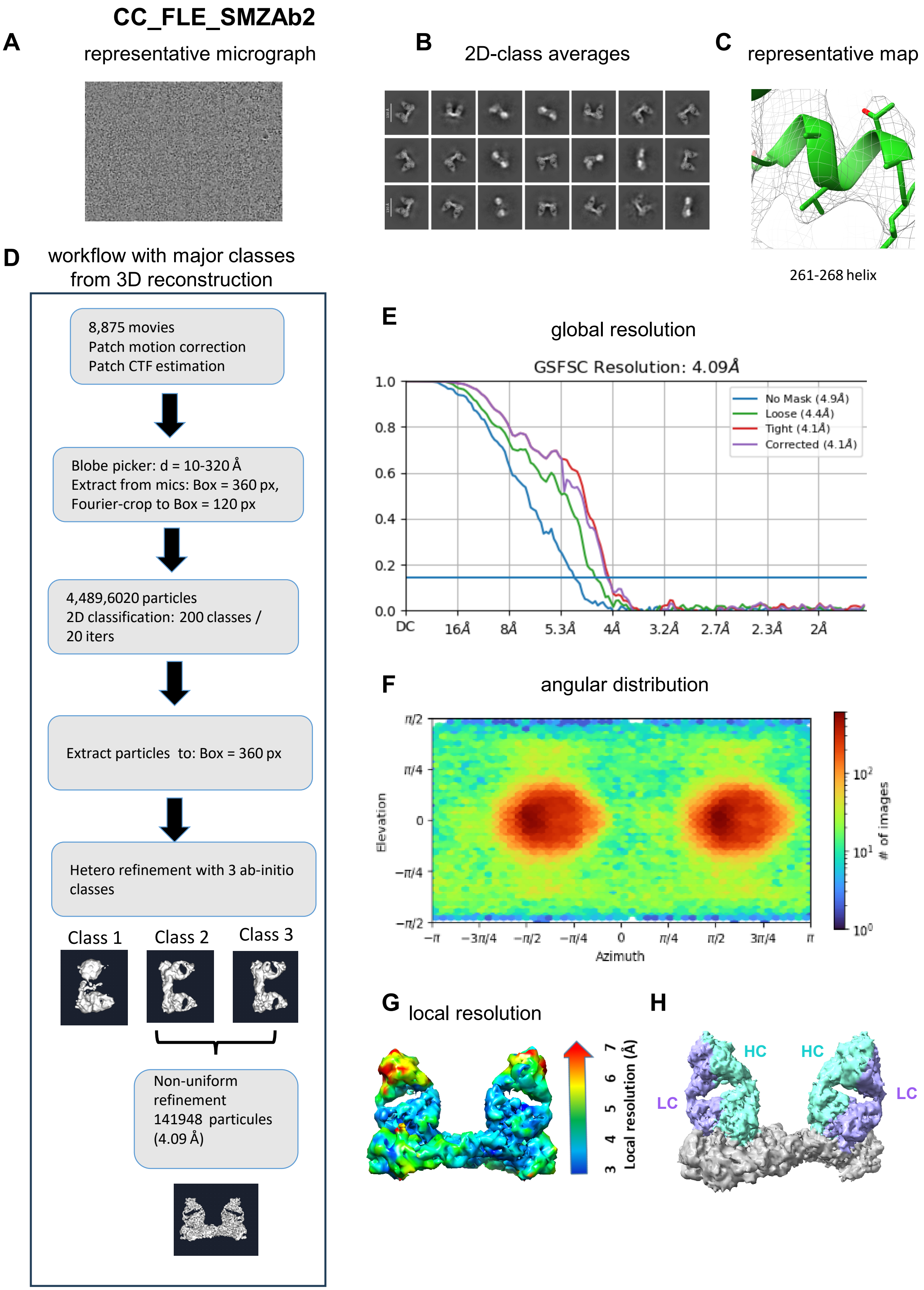

CC_FLE_SMZAb2
A
representative micrograph
B
2D-class averages
C
representative map
261-268 helix
D
workflow with major classes from 3D reconstruction
8,875 movies
Patch motion correction
Patch CTF estimation
Blobe picker: d = 10-320 Å
Extract from mics: Box = 360 px,
Fourier-crop to Box = 120 px
4,489,6020 particles
2D classification: 200 classes / 20 iters
Extract particles to: Box = 360 px
Hetero refinement with 3 ab-initio classes
Class 1
Class 2
Class 3
Non-uniform refinement
141948 particles
(4.09 Å)
E
global resolution
GSFSC Resolution: 4.09Å
No Mask (4.9Å)
Loose (4.4Å)
Tight (4.1Å)
Corrected (4.1Å)
1.0
0.8
0.6
0.4
0.2
0.0
DC
16Å
8Å
5.3Å
4Å
3.2Å
2.7Å
2.3Å
2Å
F
angular distribution
Elevation
π/2
π/4
0
−π/4
−π/2
−π
−3π/4
−π/2
−π/4
0
π/4
π/2
3π/4
π
Azimuth
# of images
10^2
10^1
10^0
G
local resolution
Local resolution (Å)
3
4
5
6
7
H
HC
HC
LC
LC

Fig. S2 (continued)

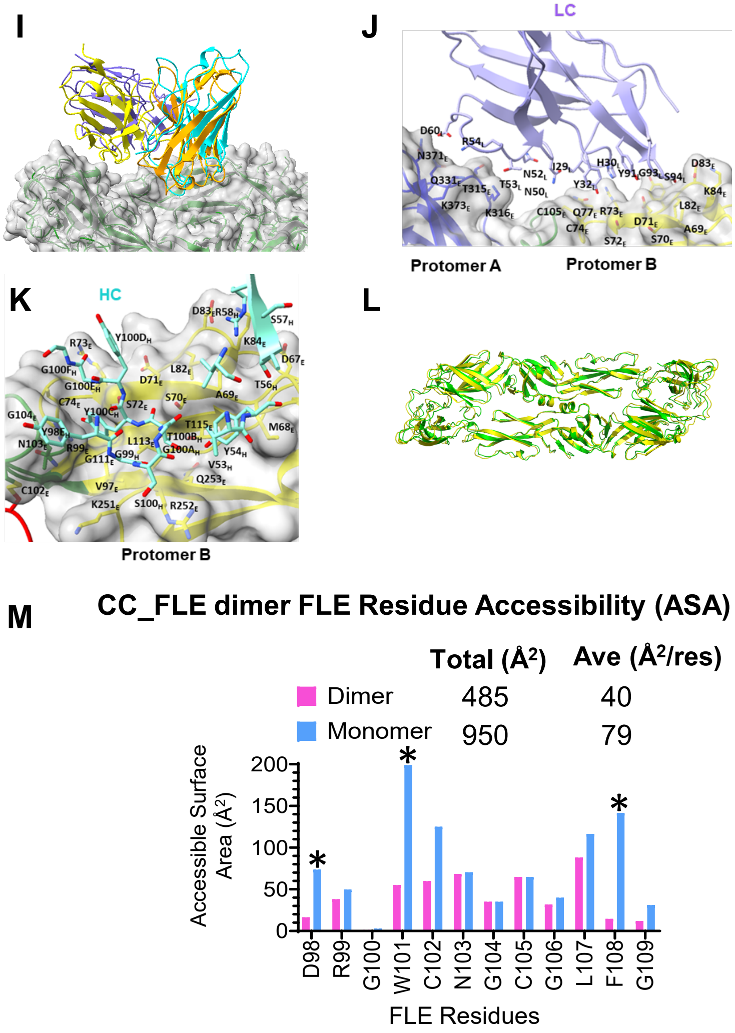

Fig. S2. Cryo-EM data of ZIKV CC_FLE sE: SMZAb2 complex data processing workflow. (A,B,C) The representative raw cryo-EM image, 2D classes and a map for 261-268 helix residues with the structural model **(D)** Cryo-EM data processing procedure workflow with representative images of major classes from 3D-reconstructions. **(E-F)** FSC curves with global resolution estimate and an angular distribution plot of particles used in the final reconstruction, (**G**) **D**ensity map colored by local resolution. (**H**) Final 4.09 Å resolution map colored by chains of bound SMZAb2 fabs: heavy chain (cyan), light chain (magenta). Density corresponding ZIKV CC_FLE sE dimer is shown in grey. (**I**) SMZAb2 shares a similar sE binding mode with EDE1-C8, as supported by structural superposition of the WT sE dimer (PDB: 5LBS, in complex with EDE1-C8) and the stabilized CC_FLE dimer in complex with the SMZAb2 Fab. The WT sE is shown as a semi-transparent gray surface with cartoon representation. The CC_FLE dimer is depicted in green. Fab fragments are colored as follows: SMZAb2 heavy and light chains colors as in (H), EDE1-C8 heavy and light chains in gold and yellow, respectively. The superposition highlights the conserved quaternary epitope geometry and binding orientation of both EDE-class antibodies. (**J-K**) Cartoon representation of interacting residues of the SMZAb2 light and heavy chains with ZIKV CC_FLE sE dimer, FLE shown in green. (**L**) Superposition of ZIKV CC_FLE dimer sE structure (yellow) with the ZIKV WT dimer sE (pdb 5lbs, green): 751 CA atoms of both chains were aligned with RMSD 1.72 Å. (**M**). Accessible surface area (ASA) of each FLE residue and the whole FLE in ZIKV CC_FLE sE assessed with PDBePISA. Residues with >50% of this ASA change are denoted with an asterisk. Ave (Å²/res), average ASA of each FLE residue. Related to **Fig. 2**.

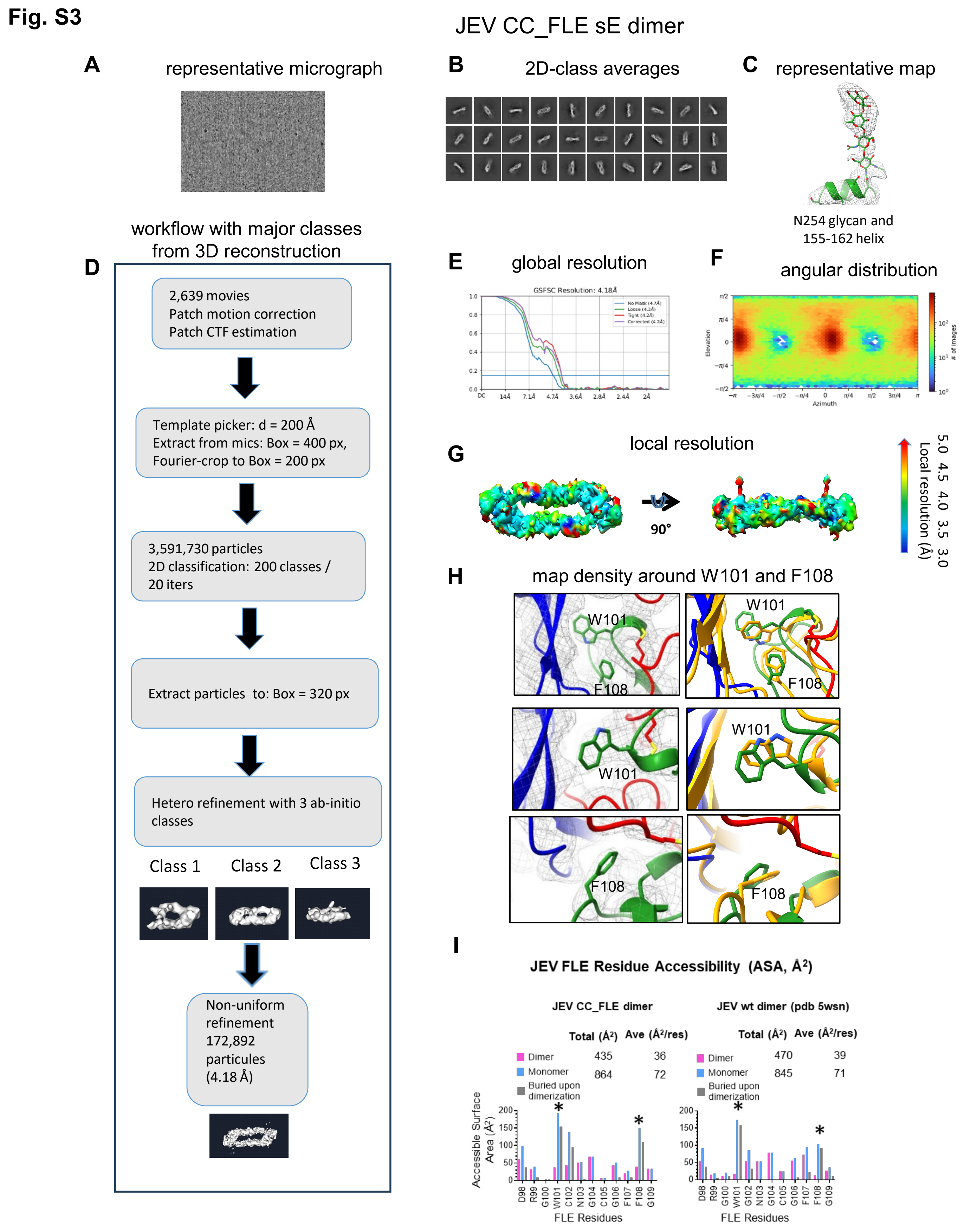
Fig. S3
JEV CC_FLE sE dimer
A
representative micrograph
B
2D-class averages
C
representative map
N254 glycan and
155-162 helix
D
workflow with major classes
from 3D reconstruction
2,639 movies
Patch motion correction
Patch CTF estimation
Template picker: d = 200 Å
Extract from mics: Box = 400 px,
Fourier-crop to Box = 200 px
3,591,730 particles
2D classification: 200 classes /
20 iters
Extract particles to: Box = 320 px
Hetero refinement with 3 ab-initio
classes
Class 1
Class 2
Class 3
Non-uniform
refinement
172,892
particules
(4.18 Å)
E
global resolution
GSFSC Resolution: 4.18Å
No Mask (4.7Å)
Loose (4.3Å)
Tight (4.2Å)
Corrected (4.2Å)
F
angular distribution
Elevation
Azimuth
# of images
G
local resolution
90°
Local resolution (Å)
5.0 4.5 4.0 3.5 3.0
H
map density around W101 and F108
W101
F108
I
JEV FLE Residue Accessibility (ASA, Å²)
JEV CC_FLE dimer
Total (Å²) Ave (Å²/res)
Dimer 435 36
Monomer 864 72
Buried upon dimerization
JEV wt dimer (pdb 5wsn)
Total (Å²) Ave (Å²/res)
Dimer 470 39
Monomer 845 71
Buried upon dimerization
Accessible Surface Area (Å²)
D98 R99 G100 W101 C102 N103 G104 C105 G106 F107 F108 G109
FLE Residues

Fig. S3 (continued)

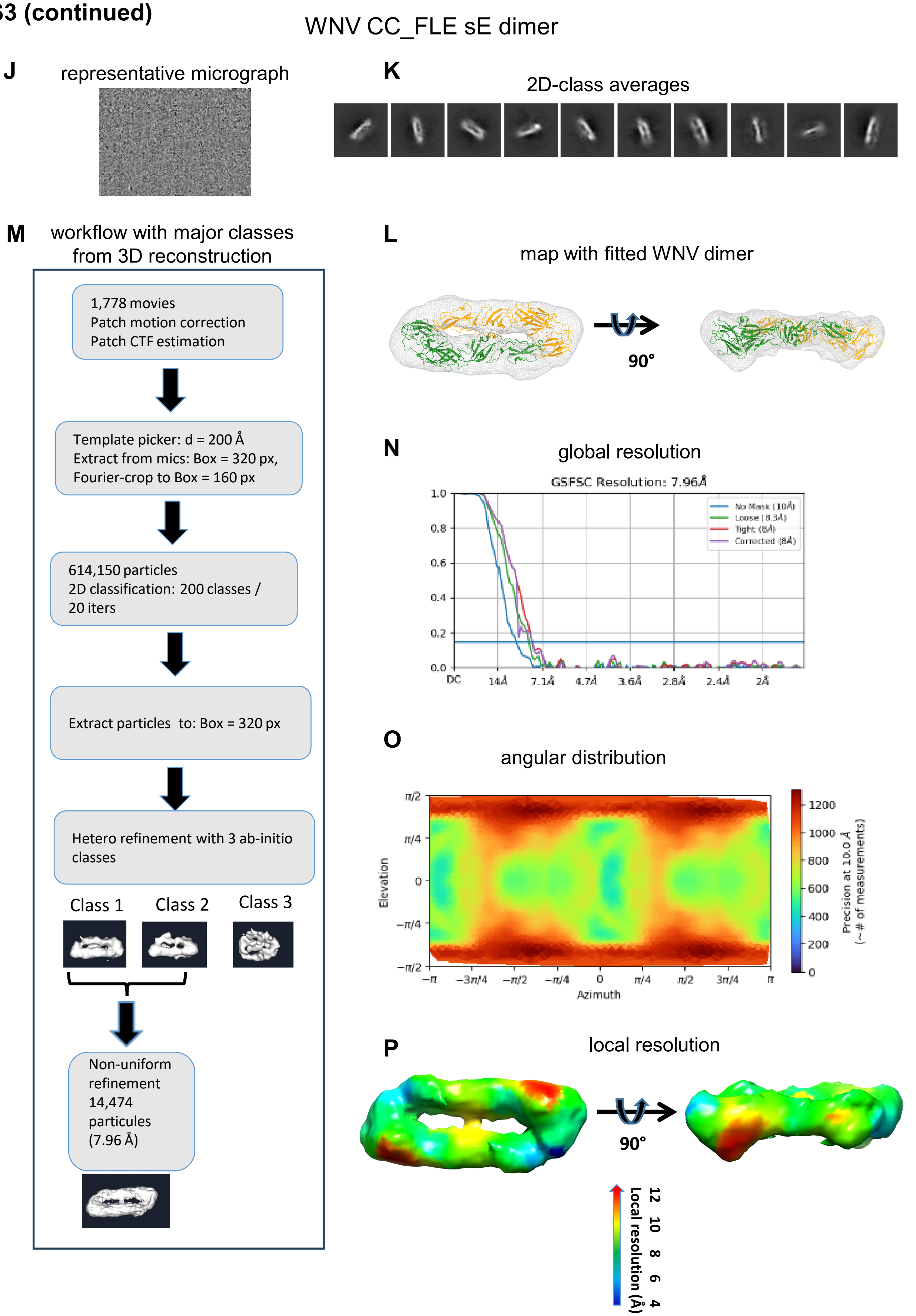

Fig. S3. Cryo-EM data processing workflow and structural details of unliganded JEV (A-I) and WNV (J-P) CC-FLE sE dimer. (**A-C**) The representative raw cryo-EM image, 2D classes and a map for 155-162 helix with adjacent N254 glycan (**D**) Cryo-EM data processing procedure workflow with representative images of major classes from 3D-reconstructions. (**E-F**) FSC curves with global resolution estimate reported at FSC 0.143 cut-off and an angular distribution plot of particles used in the final reconstruction. (**G**) Local resolution estimation of JEV CC_FLE sE dimer. (**H**) FLE residues (green) in JEV CC-FLE sE dimer context are occluded by DI (red) and DIII (blue) from adjacent protomer at the dimer interface (map density shown as a mesh), W101 and F108 (upper), W101 (middle), F108 (lower). Left: JEV CC-FLE sE dimer. Right: JEV CC-FLE sE dimer superposition with JEV wt sE dimer (pdb 5wsn). (**I**) Accessible surface area (ASA) of each FLE residue and the entire FLE in JEV sE assessed with PDBePISA is lower in the dimer context than the monomer context. W101 and F108 residues with >70% of reduction in ASA are denoted with an asterisk. Left: JEV CC-FLE sE dimer. Right: JEV wt sE dimer shown in orange color (pdb 5wsn). (**J-L**) The representative raw cryo-EM image, 2D classes and a map with fitted dimer model (PDB: 3IYW) (CC chimera 0.836) (**M**) Cryo-EM data processing procedure workflow with representative images of major classes from 3D-reconstructions. (**N-O**) FSC curves with global resolution estimate reported at FSC 0.143 cut-off and an angular distribution plot of particles used in the final reconstruction. (**P**) Local resolution estimation of WNV CC_FLE sE dimer map. Related to **Fig. 3**.

Fig. S4

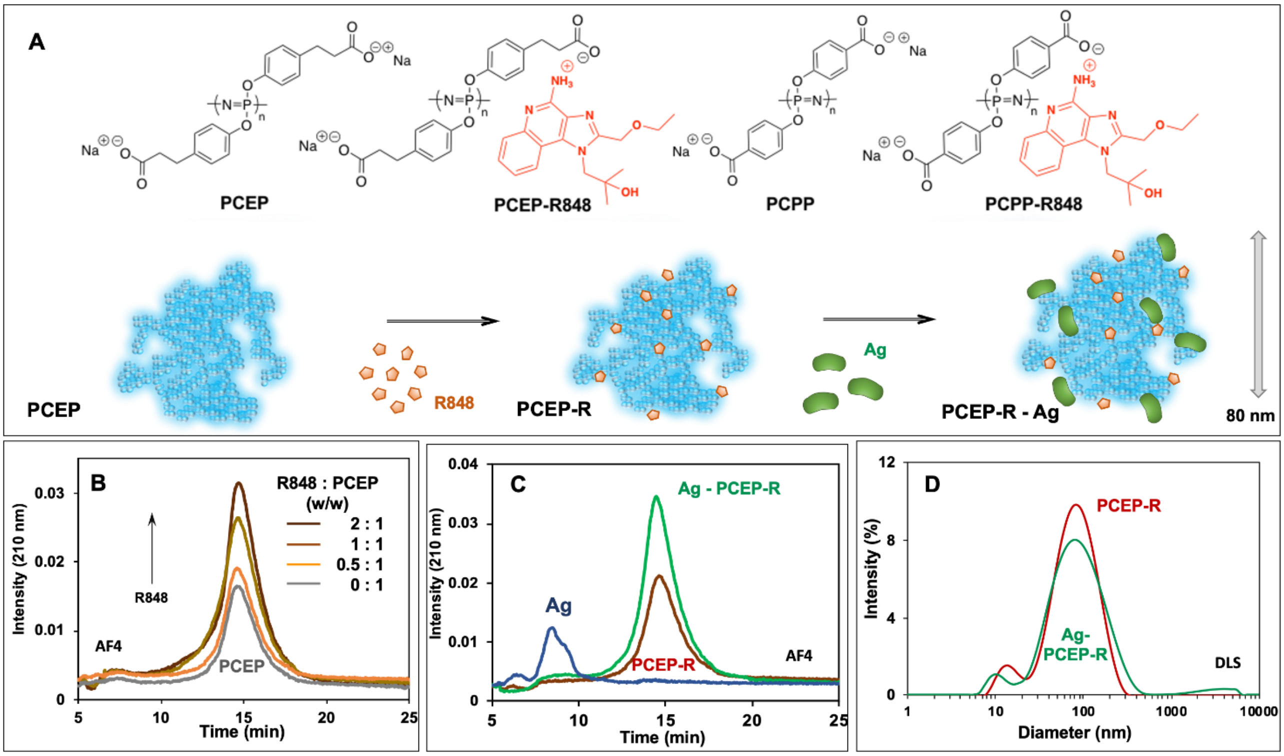

Fig. S4 . Self-assembly of virus-mimicking supramolecular complexes of CC_FLE sE-PCEP-R848. (**A**) Chemical structures of PPZ adjuvants and schematic presentation of the formation of CC_FLE sE-PCEP-R848 complex. (**B**) self-assembly of the binary PCEP-R848 complex shown by Asymmetric Flow Field Flow Fractionation (AF4) assay, with various R848: PCEP ratios (R848 is detected by AF4 only when complex with the polymer; an increase in peak area confirms R848 binding to the polymer). (**C**) self-assembly of the ternary CC_FLE sE (Ag)-PCEP-R848 complex shown by AF4 (AF4 parameters: 0.125 mg/mL PCEP, 0.0625 mg/mL R848, 0.05 mg/mL CC_FLE; PBS, 100 µL injection; pH 7.4). The AF4 fractogram of CC_FLE sE-PCEP-R848 formulation shows the disappearance of the peak corresponding to the elution time of unbound CC_FLE sE (9 min), indicating that CC_FLE sE is completely bound to the polymer in the formulation. (**D**) Self-assembly of the CC_FLE sE (Ag)-PCEP-R848 complex shown by Dynamic Light Scattering (DLS). A unimodal size distribution with a z-average hydrodynamic diameter of 60 nm confirms the nano-scale dimensions of the complex. Related to **Fig. 5**.

Fig. S5

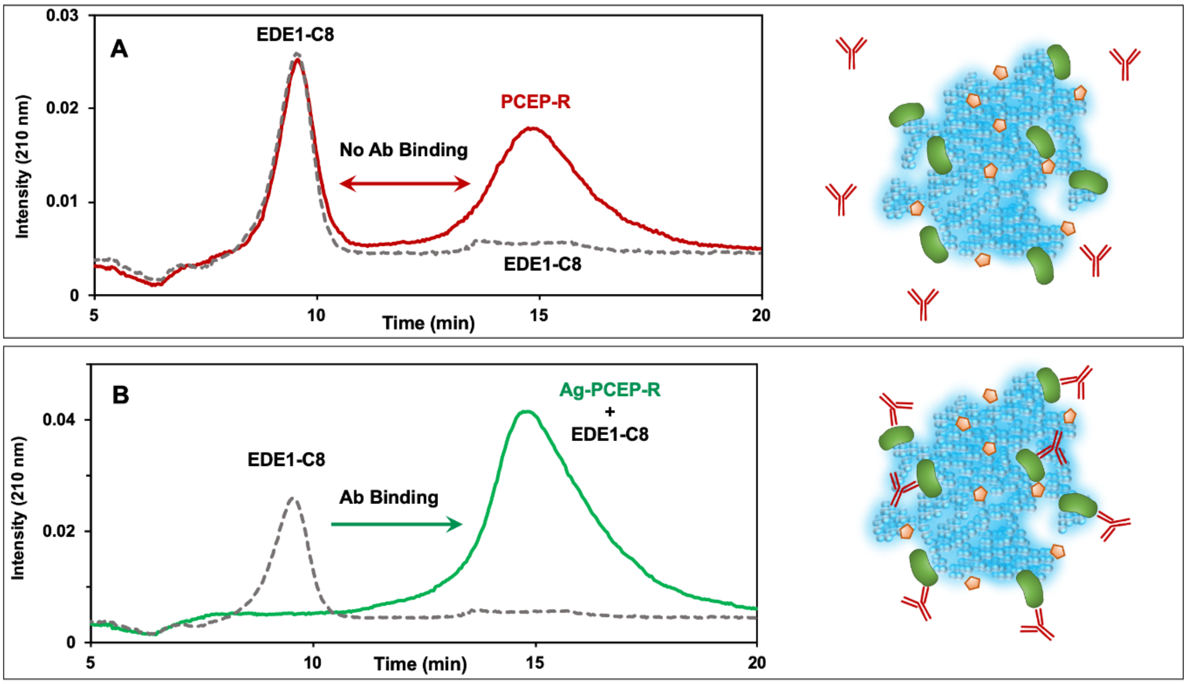

Fig. S5. **Antigenicity validation of CC_FLE sE-PCEP-R848 complex**. The CC_FLE-PCEP-R848 complex was mixed with EDE1-C8 mAb and analyzed by AF4 assay. (**A**) AF4 profile of PCEP-R848 complex and EDE1-C8 demonstrates a lack of component interactions; (**B**) AF4 profile of the CC_FLE-PCEP-R848 complex and EDE1-C8 after mixing demonstrates binding of EDE1-C8 to CC_FLE encapsulated in the ternary CC_FLE-PCEP-R848 complex, evident from the disappearance of the EDE1-C8 peak (9 min). The profile of EDE1-C8 alone is overlaid for comparison (AF4 parameters: 0.125 mg/mL PCEP, 0.0625 mg/mL R848, 0.05 mg/mL CC_FLE, 0.05 mg/ml EDE1-C8; PBS, 100 µL injection; pH 7.4). Related to **Fig. 5**.

Fig. S6

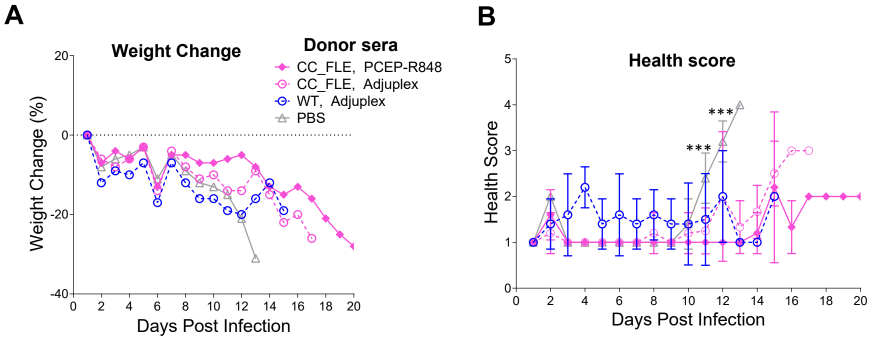

Fig. S6. Protection efficacy of passive transferred immune sera. AG129 mice (n=6/ group) were infused with 200 µl of immune sera collected on day 42 (**Fig. 4A**) via intraperitoneal route. One hour after the serum transfer, mice were challenged with ZIKV FSS13025 (1×10^4 PFU) via subcutaneous injections. (**A**) weight change and (**B**) health score of AG129 mice were monitored. Statistical analysis was performed using multiple t test (****p*<0.001) at days 11 (p=0.00045) and 12 (p=0.000045) post-ZIKV infection, comparing health scores between donor sera from PBS-inoculated mice and those inoculated with CC_FLE sE formulated in PCEP-R848. Source data are provided as a Source Data file. Related to **Fig. 5D**.

Fig. S7

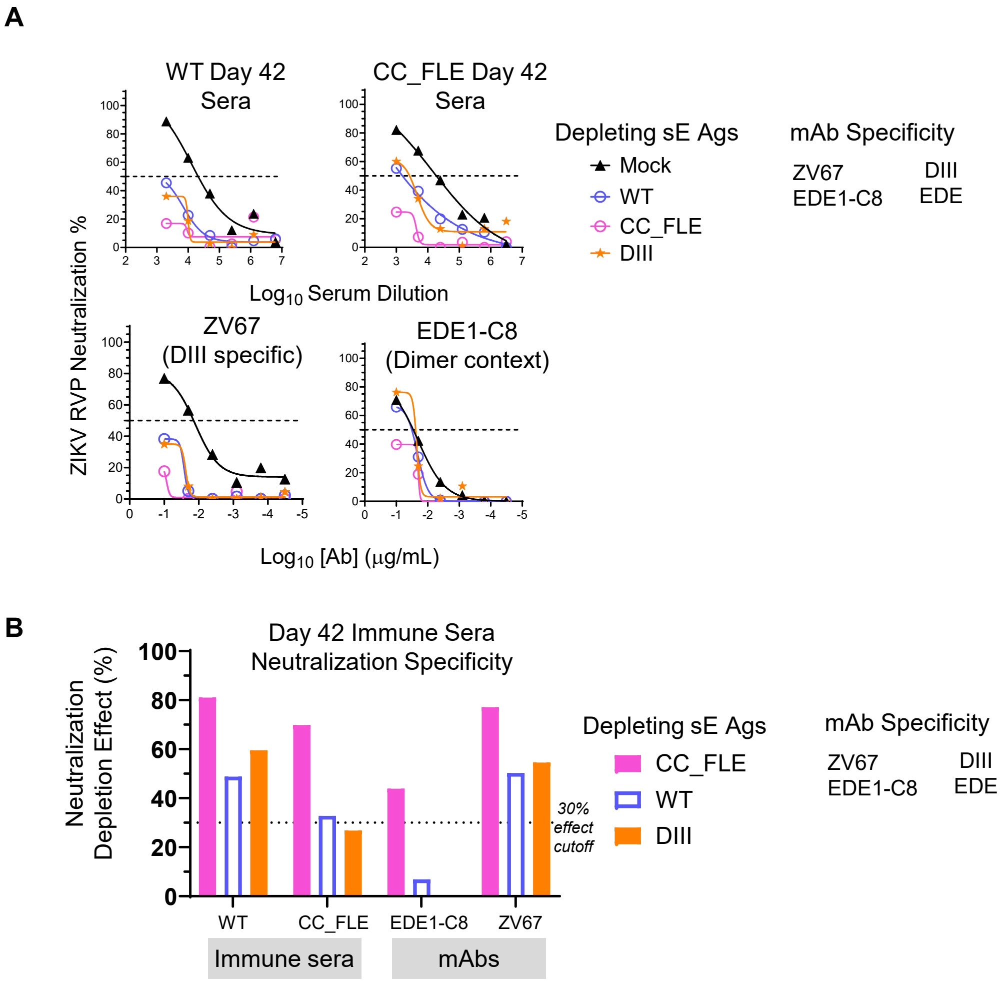

Fig. S7. Dimeric CC_FLE sE elicits neutralizing antibody response to epitopes distinctive from monomeric WT sE. (A) Immune sera neutralization activity depletion by various sE-based antigens (sE Ags). Pooled day 42 sera (**Fig. 5A**) from mice immunized with WT or CC_FLE sE immunogen formulated with Adjuplex were pre-mixed with sE variant antigens to deplete serum neutralizing antibodies prior to ZIKV H/PF/2013 RVP incubation. mAbs of ZV67 (DIII specific) and EDE1-C8 (dimer specific) were used as controls. Mock treatment, medium containing no antigen, was added to immune sera or mAb for comparison. (**B**) Summary of depletion effect of sE Ags on the neutralization activity of immune sera or mAbs. The Neutralization Depletion Effect of an Ag on each immune serum was calculated as 100*(1-(Maximal Neutralization Percentage_Ag/Maximal Neutralization Percentage_Mock)), while the effect of an Ag on each mAb, was calculated as 100*(1-(Maximal Neutralization Percentage_Ag/Maximal Neutralization Percentage_Mock)). Each sample test was duplicated, and each assay was repeated at least two times. Source data are provided as a Source Data file. Related to **Fig. 5.**

Fig. S8

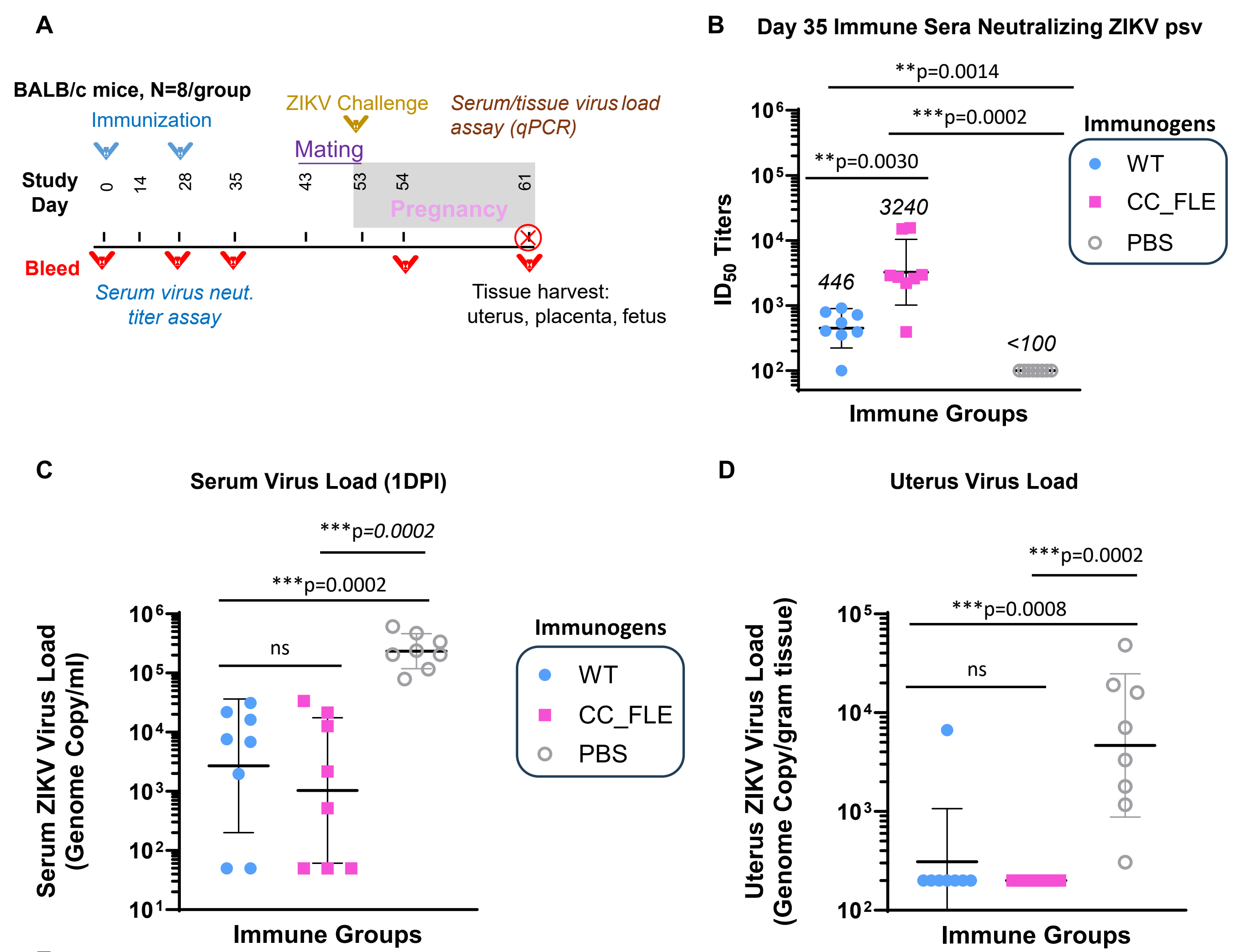

E

Animal group (immunogen)	Pregnant mice (frequency, %)	ZIKV virus load (ZIKV RNA copies/g tissue or ml blood) geomean			
		Serum	Uterus	Placenta	Fetus
WT (N=8)	5 (63%)	2.7E+03 (**p<0.01)	3.1E+02 (***p<0.001)	<200	<50
CC_FLE (N=8)	3 (38%)	1.0E+03 (***p<0.001)	<200 (****p<0.0001)	<200	<50
PBS (N=8)	0 (0%)	2.3E+05	4.7E+03	-	-

Fig. S8. Pregnant mice/fetus protection efficacy. (**A**) Immunization/challenge schedule. Female BALB/c mice (N=8/group) received 20 μg of immunogen formulated PCEP+R848 via subcutaneous route on study days 0 and 28, followed by establishment of pregnancy, and challenge with ZIKV Puerto Rico 2015 2x10^6 $TCID_{50}$/dose on day 53 via retro-orbital route. (**B**) Day 35 sera ID_{50} titers against ZIKV H/PF/2013 RVP. Each sample test was duplicated, and each assay was repeated at least two times. (**C**) Day 54 serum (1 day post challenge, 1 DPI) and (**D**) uterus virus load determination. Lines and error bars show geometric mean ± standard deviation. (**E**) Summary of pregnant mice frequency, and tissue virus load in each immunization group. No ZIKV detected in uterus, placenta or fetus of mice immunized with CC_FLE. Statistical analyses of ID_{50} titers or viral loads between PBS- and WT- or CC_FLE-vaccinated mice are performed using a two-tailed Mann–Whitney U test with a 95% confidence level. (***p*<0.01; ****p*<0.001; ****p<0.0001; ns, not significant). In the assays in (C), (D), and (E), each sample test was duplicated. Source data are provided as a Source Data file. Related to **Fig. 6**.

Fig. S9

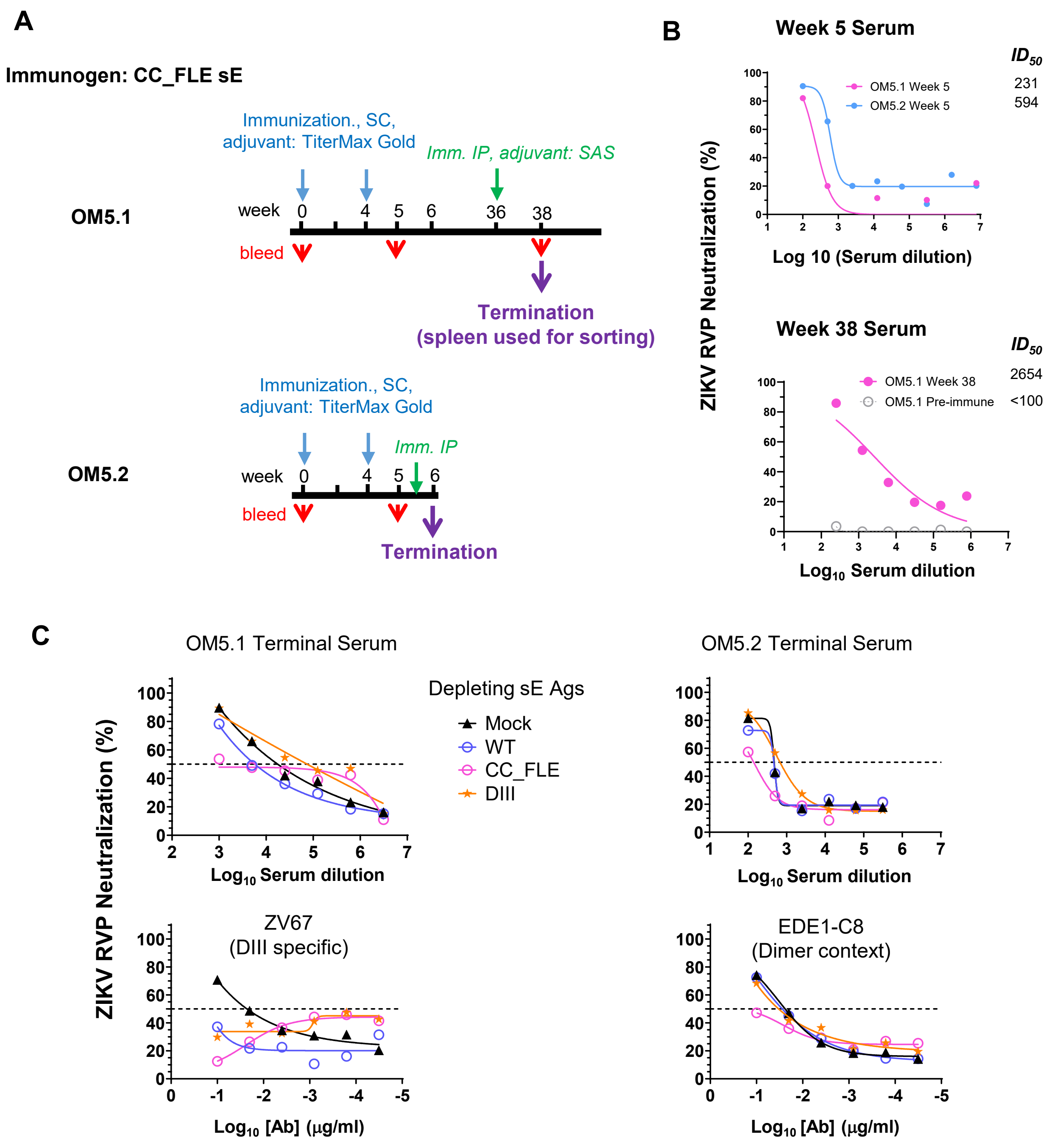
A
Immunogen: CC_FLE sE
OM5.1
Immunization., SC, adjuvant: TiterMax Gold
Imm. IP, adjuvant: SAS
week 0 4 5 6 36 38
bleed
Termination
(spleen used for sorting)
OM5.2
Immunization., SC, adjuvant: TiterMax Gold
Imm. IP
week 0 4 5 6
bleed
Termination
B
ZIKV RVP Neutralization (%)
Week 5 Serum
ID50
OM5.1 Week 5 231
OM5.2 Week 5 594
Log 10 (Serum dilution)
Week 38 Serum
ID50
OM5.1 Week 38 2654
OM5.1 Pre-immune <100
Log10 Serum dilution
C
ZIKV RVP Neutralization (%)
OM5.1 Terminal Serum
Depleting sE Ags
Mock
WT
CC_FLE
DIII
Log10 Serum dilution
OM5.2 Terminal Serum
Log10 Serum dilution
ZV67
(DIII specific)
Log10 [Ab] (μg/ml)
EDE1-C8
(Dimer context)
Log10 [Ab] (μg/ml)

Fig. S9. Immunogenicity of ZIKV CC_FLE sE in OmniMouse animals expressing naïve human immunoglobulin loci. (**A**) Immunization schedule. Two female OmniMouse mice, OM5.1 and OM5.2, aged 20-21 weeks, were immunized with 20 µg of CC_FLE sE formulated in TiterMax Gold adjuvant via subcutaneous (SC) route on weeks 0, and 4. OM5.1 received an additional immunization with 20 µg of CC_FLE sE formulated in Sigma Adjuvant System (SAS) via intraperitoneal (IP) route on week 36 and was terminated on week 38. OM5.2 was terminated on week 6 after receiving an additional immunization with 20 µg of CC_FLE sE without any adjuvant via IP route four days before week 6. (**B**) OmniMouse sera (weeks 5 and 38) neutralization activity against ZIKV H/PF/2013 RVP, with pre-immune serum as a negative control. (**C**) Immune sera neutralization activity depletion by various sE-based antigens (sE Ags). Terminal sera were pre-mixed with sE variant antigens to deplete cognate serum neutralizing antibodies prior to ZIKV H/PF/2013 RVP incubation, with mAbs ZV67 (DIII-specific) and EDE1-C8 (dimer-specific) as controls. Mock treatment, medium containing no antigen, was added to immune sera or mAb for comparison. In (B) and (C), each sample test was duplicated and each assay was repeated at least two times. Source data are provided as a Source Data file. Related to **Fig. 7**.

Fig. S10

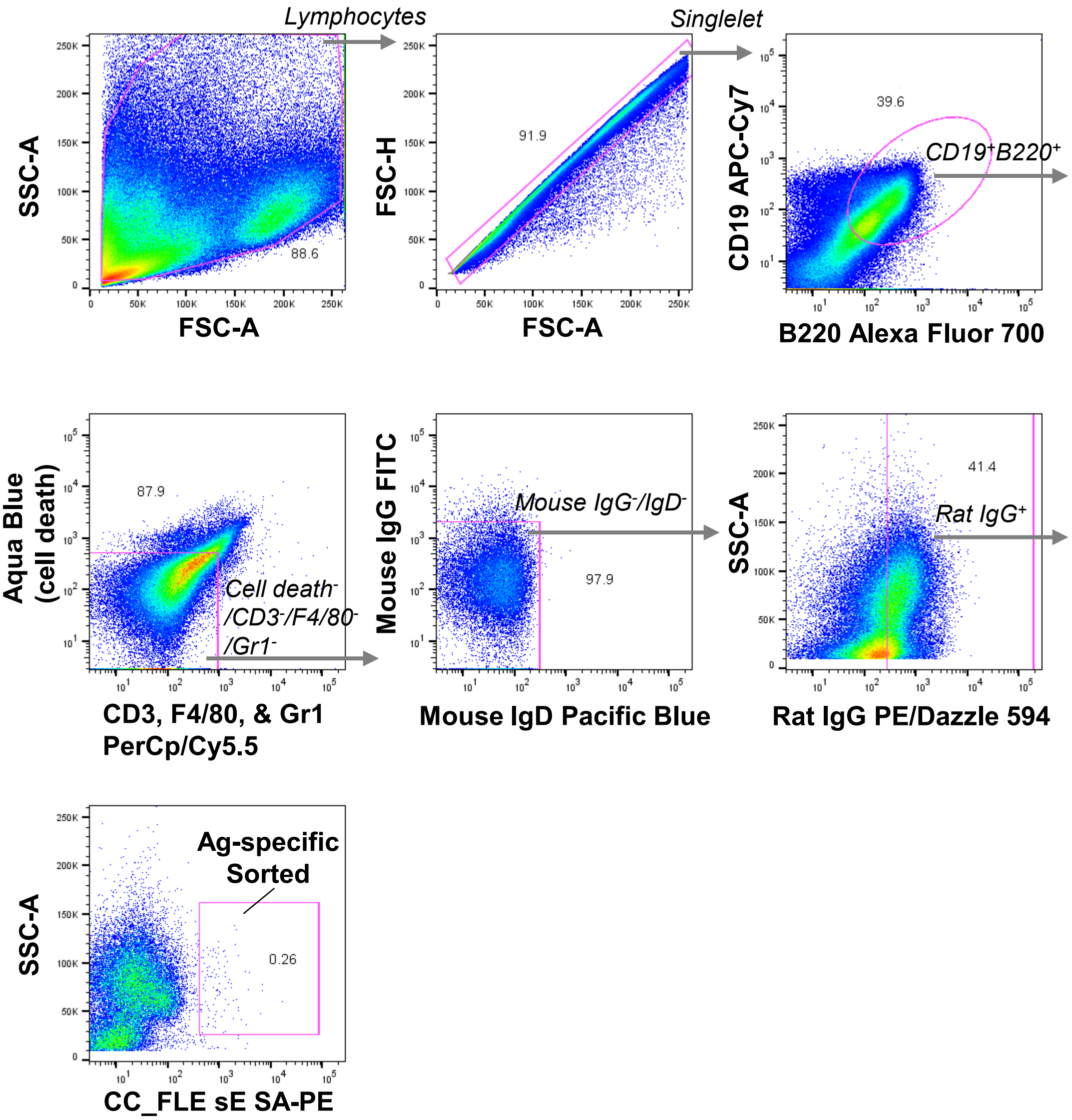

Fig. S10. Single-cell sorting of ZIKV antigen-specific B cells from splenocytes of immunized OmniMouse OM5.1. After the gating of lymphocytes (SSC-A vs. FSC-A) and singlets (FSC-H vs. FSC-A), B cells were gated with CD19$^+$/B220$^+$ phenotype, followed by negative phenotype of aqua blue and CD3/Gr1/F4/80 staining to remove dead cells and trace T cells, macrophages, or granulocytes. Antigen-specific class-switched B cells were then identified using the phenotype mouse IgD$^-$IgG$^-$/rat IgG$^+$/CC_FLE sE$^+$. The frequency (percentage) of gated cells in the corresponding parent cell population is shown. Related to **Fig. 7C**.

Fig. S11.

ZIKV E dimer CC_Core with OZ-D4

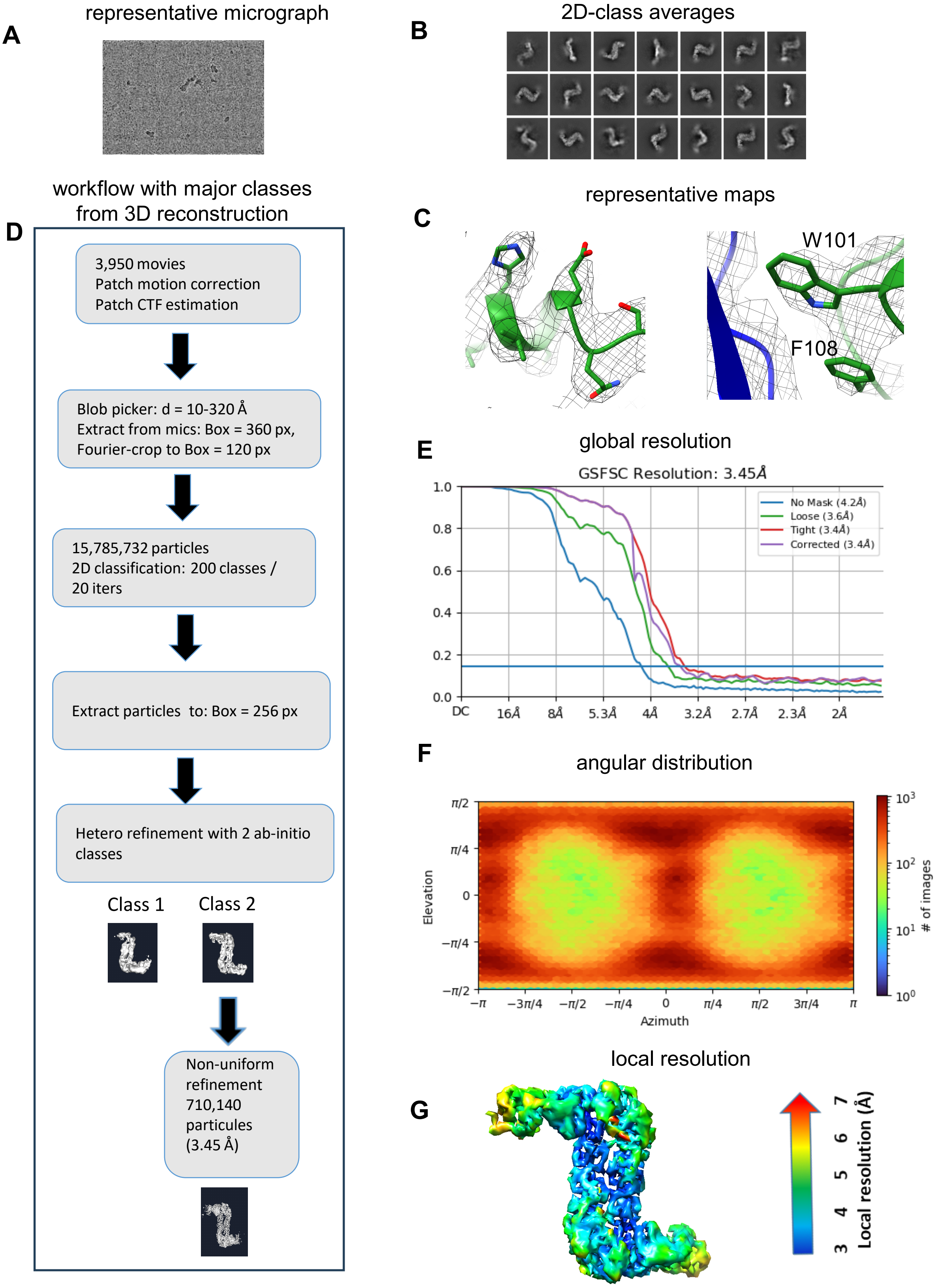

Fig. S11. Cryo-EM data processing workflow of ZIKV E dimer CC_Core with OZ-D4. (**A-B**)The representative raw cryo-EM image and 2D classes. (**C**) density maps with fitted dimer models of helix 261-268 (left) and FLE region around W101 and F1108 residues (right). (**D**) Cryo-EM data processing procedure workflow with representative images of major classes from 3D-reconstructions. (**E-F**) FSC curves with global resolution estimate reported at FSC 0.143 cut-off and an angular distribution plot of particles used in the final reconstruction. (**G**) Local resolution estimation map of ZIKV E dimer CC_Core complex with OZ-D4 Fab. Related to **Fig. 8.**

Table S1. Cryo-EM data collection, refinement, and validation statistics for ZIKV, JEV and WNV CC_FLE sE dimers

**Structure**	**ZIKV CC_FLE sE: SMZAb2**	**JEV CC_FLE sE**	**WNV CC_FLE sE**
**Data Deposition**			
**PDB ID**	9OD2	9PL9	
**EMD ID**	70338	71715	71727
**Data collection**			
Magnification	56,200x	56,200x	56,200x
Voltage (kV)	200	200	200
Microscope	Glacios	Glacios	Glacios
Electron exposure (e–/Å^2)	46.2	46.2	44,77
Defocus range (mm)	-1.0 to -2.6	-1.0 to -2.6	-1.0 to -2.6
Pixel size (Å)	0.889	0.889	0.889
Symmetry imposed	C2	C2	C2
Final particle images (no.)	141,948	172,829	14,474
Map resolution (Å)	4.09	4.18	7.96
FSC threshold	0.143	0.143	0.143
**Refinement (Phenix)**			
Model resolution (Å)	4.05	4.13	
FSC threshold	0.143	0.143	
Map sharpening B factor (Å^2)	-198.6	-183.1	
**Model composition**			
Non-hydrogen atoms (no.)	9,304	6,160	
Protein residues (no.)	1,222	802	
BMA		2	
NAG		4	
MAN		2	
**B factors**			
Protein (Å2)	127.28	98.56	
Ligand (Å2)		79.82	
R.m.s. deviations			
Bond lengths (Å)	0.003	0.004	
Bond angles (°)	0.652	0.703	
Model to map fit			
CC (mask)	0.73	0.73	
Validation			
EMRinger	1.05	1.24	
MolProbity score	1.53	1.26	
Clashscore	5.56	5.03	
Poor rotamers (%)	0.1	0.15	
**Ramachandran plot**			
Favored (%)	96.49	97.99	
Allowed (%)	3.51	2.01	
Disallowed (%)	0	0	

Table S2. Comparison of structural parameters of ZIKV and JEV sE dimers in this study and previously published work

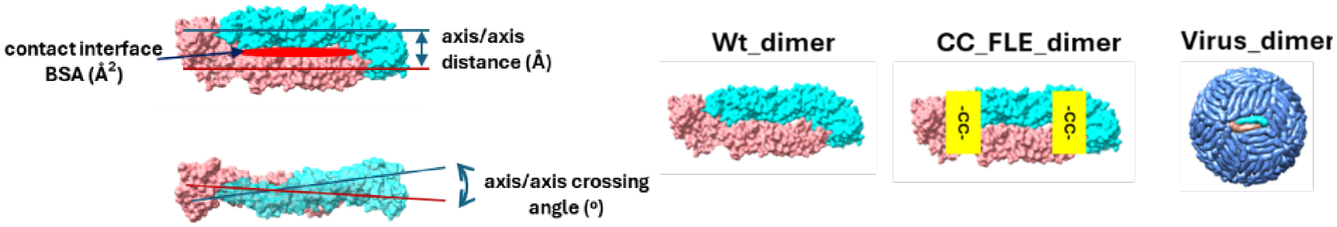

Virus	PDB ID / Name	Method / Res.	Antibody	State	Axis/Axis Distance (Å)	Axis/Axis Crossing Angle (°)	Interface BSA (Å²)
ZIKV	**7A3N**	X-ray / 2.41 Å	EDE1 C10	WT Dimer	24.2	4.0	1484.2
ZIKV	**5LBS**	X-ray / 2.10 Å	EDE1 C8	WT Dimer	24.6	9.9	1498.6
ZIKV	**5LCV**	X-ray / 2.64 Å	EDE2 A11	WT Dimer	24.5	13.3	1449.9
ZIKV	**WT Dimer Avg.**	—	—	WT Dimer	24.43 ± 0.17	9.07 ± 3.84	1477.6 ± 20.4
ZIKV	**7KCR**	CryoEM / 4.0 Å	ADI30056	Virus Dimer	24.6	11.1	1765.9
ZIKV	**7JYI**	CryoEM / 3.4 Å	None	Virus Dimer	24.6	10.6	1496.8
ZIKV	**6CO8**	CryoEM / 3.1 Å	None	Virus Dimer	24.2	10.4	1593.0
ZIKV	**Virus Dimer Avg.**	—	—	Virus Dimer	24.47 ± 0.19	10.7 ± 0.29	1618.6 ± 111.3
ZIKV	**9OD2 (CC_FLE sE–SMZAb2, this study)**	CryoEM / 4.09 Å	SMZAb2	Modified Dimer	24.5	8.8	**1689.8**
JEV	**3P54**	X-ray / 2.1 Å	None	WT Dimer	26.5	5.5	842.7
JEV	**5WSN**	CryoEM / 4.3 Å	None	Virus Dimer	25.4	11.9	1129.1
JEV	**9PL9 (JEV_FLE sE, this study)**	CryoEM / 4.18 Å	None	Modified Dimer	25.4	7.4	**1191.8**

Table S3. Genetic and functional characteristics of OmniMouse-derived monoclonal antibodies

mAb ID	Heavy Chain							Light Chain							Specificity
	VDJ gene usage			HCDR3-IMGT		% SHM[a]		VJ gene usage			LCDR3-IMGT		% SHM		
	VH	D	JH	Sequence	Length	nt	AA	VL	VK	J	Sequence	Length	nt	AA	
OZ-A1	4.34	3	6	ARGGYYYGSGSSLYYYYSGMDV	22	2.8	3.1		3.15	4	QQYNNWPLT	9	0.7	0.0	Partially DIII
OZ-B9	3.43	5	4	AKDIGYGGYGGFDY	14	4.5	9.1		1.9	2	QQLNSYPYT	9	0.4	1.0	DIII
OZ-B11	4.31	3	4	ARANYDILTGFDY	13	1.4	3.0		3.15	4	QQYNNWPFT	9	0.0	0.0	E-dimer
OZ-D4	4.59	6	3	ARYSSGWSFSFDI	13	0.0	0.0	2.23		3	SSYAGGNTLV	10	2.8	8.1	E-dimer

[a]SHM is defined as the divergence of a given mAb heavy/light chain gene segment compared with the germline version in the IMGT reference database.

Table S4. Cryo-EM data collection, refinement, and validation statistics for the ZIKV CC_Core sE: OZ-D4 Fab complex

**Data Deposition**	
**PDB ID**	9PM6
**EMD ID**	71728
**Data collection**	
Magnification	45,000x
Voltage (kV)	200
Microscope	FEI Glacios
Electron exposure (e–/Å^2)	50.8
Defocus range (mm)	-1.0 to -2.6
Pixel size (Å)	0.889
Symmetry imposed	C1
Final particle images (no.)	710,140
Map resolution (Å)	3.45
FSC threshold	0.143
**Refinement (Phenix)**	
Model resolution (Å)	3.44
FSC threshold	0.143
Map sharpening B factor (Å^2)	-130.8
**Model composition**	
Non-hydrogen atoms (no.)	9162
Protein residues (no.)	1,204
**B factors**	
Protein (Å2)	116.45
R.m.s. deviations	
Bond lengths (Å)	0.003
Bond angles (°)	0.811
Model to map fit	
CC (mask)	0.78
Validation	
EMRinger	2.08
MolProbity score	1.59
Clashscore	5.26
Poor rotamers (%)	0
**Ramachandran plot**	
Favored (%)	95.49
Allowed (%)	4.51
Disallowed (%)	0

Table S5. Monoclonal antibodies used in this study

mAb	Binding sE Ags	Specificity
EDE1-C8	ZIKV (CC-FLE, ZIKV CC_Core)	EDE
SMZAb2	ZIKV CC_FLE	EDE
Z004	ZIKV (WT, CC_FLE, CC_Core)	DIII
ZV67	ZIKV (WT, CC_FLE, CC_Core)	DIII
E53	ZIKV (WT, CC_FLE, CC_Core), JEV/WNV (WT)	FLE
E16	WNV (WT, CC_FLE)	WNV DIII
2A10G6	ZIKV (WT, CC_Core), JEV/WNV (WT)	FLE
E60	ZIKV (WT, CC_Core), JEV/WNV (WT)	FLE
4G2	ZIKV (WT, CC_Core), JEV/WNV (WT)	FLE
9E10	JEV (WT, CC_FLE)	Myc tag

Table S6. Interactions of three antibodies SMZAb2, OZ-D4 and EDE1-C8 with Zika sE dimers. Contacts were calculated with MAPIYA contact map server with distance cut-off 5 Å (https://mapiya.lcbio.pl/).

Reference:
MAPIYA contact map server for identification and visualization of molecular interactions in proteins and biological complexes
Aleksandra E Badaczewska-Dawid, Chandran Nithin, Karol Wroblewski, Mateusz Kurcinski, Sebastian Kmiecik
Nucleic Acids Research, Volume 50, Issue W1, 5 July 2022, Pages W474–W482, https://doi.org/10.1093/nar/gkac307

Interactions of SMZAb2 with CC_FLE dimer

CC_FLE	SMZAb2	Possible Interaction Forces
SER_66_B	THR_56_H	electrostatic: dipole-dipole, hydrogen bond
ASP_67_B	THR_56_H	electrostatic: ion-dipole, hydrogen bond
ASP_67_B	ARG_58_H	salt bridge, hydrogen bond
ASP_67_B	SER_57_H	electrostatic: ion-dipole, hydrogen bond
MET_68_B	THR_56_H	electrostatic: dipole-dipole, hydrogen bond
MET_68_B	TYR_54_H	electrostatic: dipole-dipole, hydrogen bond, dipole-π stacking
MET_68_B	VAL_53_H	hydrophobic
ALA_69_B	TYR_54_H	hydrophobic
ALA_69_B	ARG_58_H	induction + dispersion
SER_70_B	SER_94_L	electrostatic: dipole-dipole, hydrogen bond
SER_70_B	THR_100B_H	electrostatic: dipole-dipole, hydrogen bond
SER_70_B	GLY_100A_H	induction + dispersion
SER_70_B	TYR_100C_H	electrostatic: dipole-dipole, hydrogen bond, dipole-π stacking
SER_70_B	TYR_54_H	electrostatic: dipole-dipole, hydrogen bond, dipole-π stacking
ASP_71_B	TYR_91_L	anion-π stacking, π-π stacking, electrostatic: ion-dipole
ASP_71_B	SER_94_L	electrostatic: ion-dipole, hydrogen bond
ASP_71_B	THR_100B_H	electrostatic: ion-dipole, hydrogen bond
SER_72_B	TYR_32_L	electrostatic: dipole-dipole, hydrogen bond, dipole-π stacking
SER_72_B	TYR_100D_H	electrostatic: dipole-dipole, hydrogen bond, dipole-π stacking
SER_72_B	TYR_100C_H	electrostatic: dipole-dipole, hydrogen bond, dipole-π stacking
SER_72_B	GLY_100E_H	induction + dispersion
SER_72_B	THR_100B_H	electrostatic: dipole-dipole, hydrogen bond
ARG_73_B	HIS_30_L	ionic repulsion, hydrogen bond
ARG_73_B	TYR_32_L	cation-π stacking, π-π stacking, hydrogen bond
ARG_73_B	ILE_29_L	induction + dispersion
ARG_73_B	TYR_100C_H	cation-π stacking, π-π stacking, hydrogen bond
CYS_74_B	TYR_32_L	electrostatic: dipole-dipole, hydrogen bond
CYS_74_B	TYR_100C_H	electrostatic: dipole-dipole, hydrogen bond
GLN_77_B	TYR_32_L	electrostatic: dipole-dipole, hydrogen bond, π-π stacking
GLN_77_B	ILE_29_L	induction + dispersion
GLN_77_B	HIS_30_L	electrostatic: ion-dipole, hydrogen bond, π-π stacking
LEU_82_B	SER_94_L	induction + dispersion
ASP_83_B	SER_94_L	electrostatic: ion-dipole, hydrogen bond
LYS_84_B	ARG_58_H	ionic repulsion
VAL_97_B	TYR_100C_H	hydrophobic
ARG_99_B	TYR_100C_H	cation-π stacking, π-π stacking, hydrogen bond
CYS_102_B	TYR_98_H	electrostatic: dipole-dipole, hydrogen bond
ASN_103_B	TYR_98_H	electrostatic: dipole-dipole, hydrogen bond, π-π stacking, dipole-π stacking

CC_FLE	SMZAb2	Possible Interaction Forces
ASN_103_B	TYR_100C_H	electrostatic: dipole-dipole, hydrogen bond, π-π stacking, dipole-π stacking
CYS_105_B	ASN_50_L	electrostatic: dipole-dipole, hydrogen bond
GLY_111_B	TYR_100C_H	hydrophobic
SER_112_B	TYR_100C_H	electrostatic: dipole-dipole, hydrogen bond, dipole-π stacking
LEU_113_B	TYR_100C_H	hydrophobic
LEU_113_B	THR_100B_H	induction + dispersion
THR_115_B	THR_100B_H	electrostatic: dipole-dipole, hydrogen bond
THR_115_B	TYR_54_H	electrostatic: dipole-dipole, hydrogen bond, dipole-π stacking
LYS_251_B	GLY_99_H	hydrophobic
ARG_252_B	GLY_99_H	induction + dispersion
ARG_252_B	SER_100_H	electrostatic: ion-dipole, hydrogen bond
ARG_252_B	THR_100B_H	electrostatic: ion-dipole, hydrogen bond
GLN_253_B	TYR_54_H	electrostatic: dipole-dipole, hydrogen bond, π-π stacking, dipole-π stacking
GLN_253_B	THR_100B_H	electrostatic: dipole-dipole, hydrogen bond
THR_315_A	ARG_54_L	electrostatic: ion-dipole, hydrogen bond
THR_315_A	ASN_52_L	electrostatic: dipole-dipole, hydrogen bond
LYS_316_A	THR_53_L	electrostatic: ion-dipole, hydrogen bond
GLN_331_A	ARG_54_L	electrostatic: ion-dipole, hydrogen bond
GLN_331_A	ASP_60_L	electrostatic: ion-dipole, hydrogen bond
ASN_371_A	ASP_60_L	electrostatic: ion-dipole, hydrogen bond

Interactions of OZ-D4 with CC_CORE dimer

CC_CORE	OZ-D4	Possible Interaction Type(s)
ARG:73_B	TYR:32_H	cation–π stacking, π–π stacking, hydrogen bond
CYS:74_B	TRP:99_H	hydrophobic
PRO:75_B	TRP:99_H	hydrophobic
THR:76_B	SER:31_H	electrostatic, dipole–dipole, hydrogen bond
THR:76_B	TYR:32_H	electrostatic, dipole–dipole, hydrogen bond
THR:76_B	TRP:33_H	electrostatic, dipole–dipole, hydrogen bond
THR:76_B	TYR:52_H	electrostatic, dipole–dipole, hydrogen bond
THR:76_B	SER:96_H	electrostatic, dipole–dipole, hydrogen bond
THR:76_B	TRP:99_H	electrostatic, dipole–dipole, hydrogen bond
GLN:77_B	GLY:98_H	induction + dispersion
GLN:77_B	TRP:99_H	hydrogen bond, π–π stacking
GLN:77_B	SER:31_H	electrostatic, dipole–dipole, hydrogen bond
GLN:77_B	SER:96_H	electrostatic, dipole–dipole, hydrogen bond
GLN:77_B	SER:97_H	electrostatic, dipole–dipole, hydrogen bond
GLN:77_B	SER:97_H	electrostatic, dipole–dipole, hydrogen bond
GLN:77_B	TRP:99_H	hydrogen bond, π–π stacking, dipole–π stacking
GLY:78_B	SER:30_H	induction + dispersion
GLY:78_B	TYR:32_H	induction + dispersion
GLY:78_B	TYR:32_H	hydrophobic
GLY:78_B	SER:30_H	electrostatic, ion–dipole, hydrogen bond
CYS:105_B	TRP:99_H	hydrophobic
GLY:106_B	TRP:99_H	hydrophobic

CC_CORE	OZ-D4	Possible Interaction Type(s)
LEU:107_B	TRP:99_H	hydrophobic
THR:313_A	GLY:28_L	induction + dispersion
THR:313_A	SER:29_L	electrostatic, dipole–dipole, hydrogen bond
PHE:314_A	SER:29_L	dipole–π stacking
PHE:314_A	TYR:30_L	π–π stacking, hydrophobic, dipole–π stacking
PHE:314_A	ASN:31_L	π–π stacking
THR:315_A	ASN:31_L	electrostatic, dipole–dipole, hydrogen bond
THR:315_A	LEU:32_L	induction + dispersion
ILE:317_A	TRP:99_H	hydrophobic
ILE:317_A	PHE:100_A	hydrophobic
ILE:317_A	TYR:30_L	hydrophobic
ILE:317_A	LEU:32_L	hydrophobic
ILE:317_A	TYR:91_L	hydrophobic
PRO:318_A	TYR:30_L	hydrophobic
ILE:396_A	LEU:32_L	induction + dispersion
THR:397_A	TYR:30_L	electrostatic, dipole–dipole, hydrogen bond
HIS:398_A	TYR:30_L	cation–π stacking, π–π stacking
HIS:398_A	TYR:91_L	cation–π stacking, π–π stacking, hydrogen bond

Interactions of EDE1-C8 with WT dimer (PDB 5LBS)

WT dimer	EDE1-C8	Possible Interaction Forces
ARG:2_A	ASN:98_H	electrostatic: ion-dipole, hydrogen bond
ASP:67_B	TYR:59_H	anion-π stacking, π-π stacking, electrostatic: ion-dipole, hydrogen bond
MET:68_B	ASP:55_H	electrostatic: ion-dipole
MET:68_B	SER:56_H	electrostatic: dipole-dipole, hydrogen bond
MET:68_B	ALA:57_H	hydrophobic
ALA:69_B	SER:56_H	induction + dispersion
ALA:69_B	ALA:57_H	hydrophobic
ALA:69_B	TYR:100A_H	hydrophobic
SER:70_B	SER:56_H	electrostatic: dipole-dipole, hydrogen bond
SER:70_B	ALA:57_H	induction + dispersion
SER:70_B	TYR:100_H	electrostatic: dipole-dipole, hydrogen bond, dipole-π stacking
SER:70_B	TYR:100A_H	electrostatic: dipole-dipole, hydrogen bond, dipole-π stacking
SER:70_B	TRP:94_L	hydrogen bond, dipole-π stacking
ASP:71_B	TRP:94_L	anion-π stacking, π-π stacking, hydrogen bond
SER:72_B	PHE:99_H	dipole-π stacking
SER:72_B	ASN:93_L	electrostatic: dipole-dipole, hydrogen bond
SER:72_B	TRP:94_L	hydrogen bond, dipole-π stacking
ARG:73_B	ASN:93_L	electrostatic: ion-dipole, hydrogen bond
CYS:74_B	TYR:92_L	electrostatic: dipole-dipole, hydrogen bond
CYS:74_B	ASN:93_L	electrostatic: dipole-dipole, hydrogen bond

WT dimer	EDE1-C8	Possible Interaction Forces
GLN:77_B	GLN:27_L	electrostatic: dipole-dipole, hydrogen bond
GLN:77_B	TYR:92_L	electrostatic: dipole-dipole, hydrogen bond, π-π stacking, dipole-π stacking
ASP:83_B	LYS:64_H	salt bridge, hydrogen bond
VAL:97_B	PHE:99_H	hydrophobic
ASP:98_B	PHE:99_H	anion-π stacking
ARG:99_B	PHE:99_H	cation-π stacking
ARG:99_B	ASN:93_L	electrostatic: ion-dipole, hydrogen bond
ARG:99_B	TRP:94_L	cation-π stacking
GLY:100_B	PHE:32_L	hydrophobic
TRP:101_B	TYR:100B_H	hydrogen bond, π-π stacking, dipole-π stacking
TRP:101_B	PHE:32_L	π-π stacking, hydrophobic
GLY:102_B	ASN:98_H	induction + dispersion
GLY:102_B	TYR:100B_H	hydrophobic
ASN:103_B	PHE:99_H	π-π stacking
ASN:103_B	TYR:100B_H	electrostatic: dipole-dipole, hydrogen bond, π-π stacking, dipole-π stacking
ASN:103_B	ASN:93_L	electrostatic: dipole-dipole, hydrogen bond
GLY:104_B	TYR:100B_H	hydrophobic
GLY:104_B	PHE:32_L	hydrophobic
GLY:104_B	ARG:91_L	induction + dispersion
GLY:104_B	TYR:92_L	hydrophobic
GLY:104_B	ASN:93_L	induction + dispersion
CYS:105_B	PHE:32_L	hydrophobic
CYS:105_B	TYR:92_L	electrostatic: dipole-dipole, hydrogen bond
CYS:105_B	ASN:93_L	electrostatic: dipole-dipole, hydrogen bond
GLY:106_B	SER:30_L	induction + dispersion
GLY:106_B	PHE:32_L	hydrophobic
GLY:106_B	TYR:92_L	hydrophobic
LEU:113_B	TYR:100_H	hydrophobic
LEU:113_B	TRP:94_L	hydrophobic
THR:115_B	TYR:100_H	electrostatic: dipole-dipole, hydrogen bond, dipole-π stacking
LYS:251_B	PHE:99_H	cation-π stacking
LYS:251_B	TYR:100_H	cation-π stacking, hydrogen bond
ARG:252_B	GLU:53_H	salt bridge, hydrogen bond
ARG:252_B	ASP:55_H	salt bridge, hydrogen bond
ARG:252_B	TYR:100_H	cation-π stacking, π-π stacking, hydrogen bond
GLN:253_B	TYR:100_H	electrostatic: dipole-dipole, hydrogen bond, π-π stacking, dipole-π stacking
THR:254_B	ASP:55_H	electrostatic: ion-dipole, hydrogen bond
THR:315_A	THR:31_L	electrostatic: dipole-dipole, hydrogen bond
THR:315_A	SER:52_L	electrostatic: dipole-dipole, hydrogen bond
THR:315_A	ARG:66_L	electrostatic: ion-dipole, hydrogen bond
LYS:316_A	SER:30_L	electrostatic: ion-dipole, hydrogen bond
LYS:316_A	THR:31_L	electrostatic: ion-dipole, hydrogen bond

WT dimer	EDE1-C8	Possible Interaction Forces
LYS:316_A	PHE:32_L	cation-π stacking
LYS:316_A	ARG:66_L	ionic repulsion (CAUTION: possible repulsion)
GLU:329_A	THR:31_L	electrostatic: ion-dipole, hydrogen bond
GLN:331_A	SER:52_L	electrostatic: dipole-dipole, hydrogen bond
GLN:331_A	GLY:64_L	induction + dispersion
THR:369_A	ARG:54_L	electrostatic: ion-dipole, hydrogen bond
LYS:373_A	TYR:49_L	cation-π stacking, hydrogen bond
LYS:373_A	ASP:50_L	salt bridge, hydrogen bond
LYS:373_A	SER:52_L	electrostatic: ion-dipole, hydrogen bond
LYS:373_A	THR:53_L	electrostatic: ion-dipole, hydrogen bond

The uncropped gel image in FigS1B

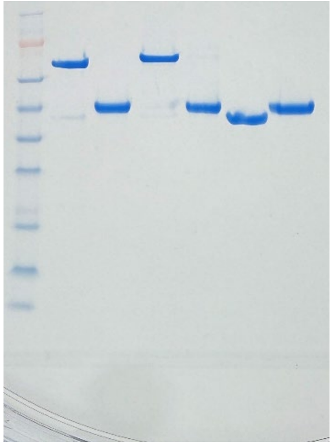

Supplement: Supplementary file 1 — Supplementary Information [file 41467_2025_67447_MOESM1_ESM.pdf]
