# Supplementary material for: Rational design of flavivirus E protein vaccine optimizes immunogenicity and mitigates antibody dependent enhancement risk
Source: Nat Commun. 2025 Dec 22;16:11558. doi: 10.1038/s41467-025-67447-4 (PMC12748774; doi:10.1038/s41467-025-67447-4)
Supplement: Supplementary file 2 — Description of Additional Supplementary Files [file 41467_2025_67447_MOESM2_ESM.pdf]

Description of Additional Supplementary Files

File Name: Supplementary Data 1
Description: Sequences of primers used for amplifying OmniMouse Ig gene segments
