# Supplementary material for: Rational design of flavivirus E protein vaccine optimizes immunogenicity and mitigates antibody dependent enhancement risk
Source: Nat Commun. 2025 Dec 22;16:11558. doi: 10.1038/s41467-025-67447-4 (PMC12748774; doi:10.1038/s41467-025-67447-4)
Supplement: Supplementary file 4 — Reporting Summary [file 41467_2025_67447_MOESM4_ESM.pdf]

nature portfolio

Corresponding author(s): Yuxing Li & Edwin Pozharski

Last updated by author(s): 11/10-2025

Reporting Summary

Nature Portfolio wishes to improve the reproducibility of the work that we publish. This form provides structure for consistency and transparency in reporting. For further information on Nature Portfolio policies, see our Editorial Policies and the Editorial Policy Checklist.

Statistics

For all statistical analyses, confirm that the following items are present in the figure legend, table legend, main text, or Methods section.

n/a	Confirmed	
☐	☒	The exact sample size (*n*) for each experimental group/condition, given as a discrete number and unit of measurement
☐	☒	A statement on whether measurements were taken from distinct samples or whether the same sample was measured repeatedly
☐	☒	The statistical test(s) used AND whether they are one- or two-sided<br>*Only common tests should be described solely by name; describe more complex techniques in the Methods section.*
☐	☒	A description of all covariates tested
☐	☒	A description of any assumptions or corrections, such as tests of normality and adjustment for multiple comparisons
☐	☒	A full description of the statistical parameters including central tendency (e.g. means) or other basic estimates (e.g. regression coefficient) AND variation (e.g. standard deviation) or associated estimates of uncertainty (e.g. confidence intervals)
☐	☒	For null hypothesis testing, the test statistic (e.g. *F*, *t*, *r*) with confidence intervals, effect sizes, degrees of freedom and *P* value noted<br>*Give P values as exact values whenever suitable.*
☒	☐	For Bayesian analysis, information on the choice of priors and Markov chain Monte Carlo settings
☒	☐	For hierarchical and complex designs, identification of the appropriate level for tests and full reporting of outcomes
☐	☒	Estimates of effect sizes (e.g. Cohen's *d*, Pearson's *r*), indicating how they were calculated

Our web collection on statistics for biologists contains articles on many of the points above.

Software and code

Policy information about availability of computer code

Data collection	BD FACSDiva software V8.02 is used to collect FACS sorting data; The Cyo-EM micrographs are collected using SerialEM software version 4.1.
Data analysis	FlowJo V10.7.2 is used to analyze FACS data;Graph Pad Prism V10.1.0 is used to perform curve fitting and statistical analysis; Disulfide bond design was conducted using Disulfide by Design 2.0; Pymol2 and UCSF Chimera version 1.18 are used to visualize protein structure; Protein buried surface areas, accessible surface areas, and residue contacts are calculated using the PDBePISA server (https://www.ebi.ac.uk/pdbe/pisa/), Chimera version 1.18, or MAPIYA (https://mapiya.lcbio.pl/); The micrographs were processed with cryoSPARC version 3.3.0. UCSF Chimera version 1.18, Coot (version 0.9.8.1), Rosetta (version 3.13), and Phenix (version 1.20) are used for protein structure building and refinement.

For manuscripts utilizing custom algorithms or software that are central to the research but not yet described in published literature, software must be made available to editors and reviewers. We strongly encourage code deposition in a community repository (e.g. GitHub). See the Nature Portfolio guidelines for submitting code & software for further information.

Data

Policy information about availability of data

All manuscripts must include a data availability statement. This statement should provide the following information, where applicable:

- Accession codes, unique identifiers, or web links for publicly available datasets
- A description of any restrictions on data availability
- For clinical datasets or third party data, please ensure that the statement adheres to our policy

The structural coordinates data and/or cryo-EM map generated in this study have been deposited in the RCSB Protein Data Bank (PDB) and the Electron Microscopy Data Bank (EMDB) database, respectively, under the following accession numbers: ZIKV CC_FLE sE:SMZAb2 complex, PDB: 9OD2, EMD: 70338; JEV CC_FLE sE, PDB: 9PL9, EMD: 71715; WNV CC_FLE sE, EMD: 71727; and ZIKV CC_Core sE:OZ-D4 Fab complex, PDB: 9PM6, EMD: 71728.
The nucleic acid sequences of OmniMouse animal-derived ZIKV mAbs have been deposited in the GenBank/NCBI database, with the following accession numbers: OZ-A1 VH, GenBank: PQ465206; OZ-A1 VK, GenBank: PQ465207; OZ-B9 VH, GenBank: PQ465208; OZ-B9 VK, GenBank: PQ465209; OZ-B11 VH, GenBank: PQ465210 ; OZ-B11 VK, GenBank: PQ465211; OZ-D4 VH, GenBank: PQ465212 ; OZ-D4 VL, GenBank: PQ465213. The other data generated in this study are provided in the Supplementary Information/Source Data file. Source data are provided with this paper.

Research involving human participants, their data, or biological material

Policy information about studies with human participants or human data. See also policy information about sex, gender (identity/presentation), and sexual orientation and race, ethnicity and racism.

Reporting on sex and gender	Not Applicable
Reporting on race, ethnicity, or other socially relevant groupings	Not Applicable
Population characteristics	Not Applicable
Recruitment	Not Applicable
Ethics oversight	Not Applicable

Note that full information on the approval of the study protocol must also be provided in the manuscript.

Field-specific reporting

Please select the one below that is the best fit for your research. If you are not sure, read the appropriate sections before making your selection.

☒ Life sciences ☐ Behavioural & social sciences ☐ Ecological, evolutionary & environmental sciences

For a reference copy of the document with all sections, see nature.com/documents/nr-reporting-summary-flat.pdf

Life sciences study design

All studies must disclose on these points even when the disclosure is negative.

Sample size

For the initial immunogen screening study in mice (Fig. 4), we included 4 female mice/group. This was based on our previous experience with immunogen screening in which N=4 is sufficient to identify the lead immunogen with biologically significant advantages over the other vaccine candidates for further analysis. The purpose is to minimize the use of animals while we are still able to identify the best immunogen to pursue further (see details for further studies in the following paragraph). The sample size was not determined by any statistical method for this screening study.
For adjuvant optimization (Fig. 5) and efficacy (Fig. 6) mouse studies, we used N=6/ female group. In determining the number of mice to be used in these experiments, we referred to "Statistical aspects of planning and design of immunological experiments", Elton, R.A. and McBride, W.H. In The Handbook of Experimental Immunology 4th edition, 1986, Vol 4, Chapter 131, pp131.1-131.6, Blackwell, Oxford. In comparing two means with similar sample sizes and normal distributions, the formula used is:
n= 20(S.D.)^2/d^2 where n, S.D., and d are the sample size, standard deviation of measurements and the differences in the experimental and control means. The referenced text notes that, for added stringency, 20 would be replaced by 30. Because in measurements of relevant parameters of immune cell function (including Ab production, specific mRNA expression, protein expression, cell proliferation, survival) the standard deviation approximates the mean, in order to detect a two-fold difference between experimental groups, a sample size of 5-7 mice is needed per group. Therefore, we used N=6/group.
For the pregnancy protection efficacy study (Fig. S8), we used n = 8 females per group. This number was based on our previously stated rationale that a sample size of 5–7 mice per group is sufficient for statistical analysis. To account for the possibility of unexpected animal loss, we included 8 mice per group to ensure adequate sample size and data integrity.
For the Omnimouse animal immunization study (Fig. 7), we used 2 female animals/group. The purpose of this study is to obtain sufficient sera and immune cells for neutralization specificity profiling and monoclonal antibody isolation. The pre-immune sera were sufficient to serve as negative control. The sample size was not determined by any statistical method for this antibody isolation study, but by the focused goal of the study.

Data exclusions	no data were excluded from the analysis
Replication	Data of assays were duplicated. Each assay was repeated at least two times to confirm the reproducibility.
Randomization	Animals were randomly assigned into different groups for all the studies.
Blinding	Animal study investigators were blinded to group allocations, as the immunogen details were not disclosed to the animal study personnel. The same with viral load determination process. For serum ELISA binding and virus neutralization assays, investigators were not blinded, as they acknowledged the grouping information. However, assays were repeated by different investigators to assure reproducibility and avoid bias.

Reporting for specific materials, systems and methods

We require information from authors about some types of materials, experimental systems and methods used in many studies. Here, indicate whether each material, system or method listed is relevant to your study. If you are not sure if a list item applies to your research, read the appropriate section before selecting a response.

Materials & experimental systems

n/a	Involved in the study
☐	☒ Antibodies
☐	☒ Eukaryotic cell lines
☒	☐ Palaeontology and archaeology
☐	☒ Animals and other organisms
☒	☐ Clinical data
☒	☐ Dual use research of concern
☒	☐ Plants

Methods

n/a	Involved in the study
☒	☐ ChIP-seq
☐	☒ Flow cytometry
☒	☐ MRI-based neuroimaging

Antibodies

Antibodies used	Plasmids encoding the heavy and light chains of antibodies EDE1-C8, SMZAb2, 2A10G6, , E16, E53, E60, Z004, Z006, and ZV67, in a human IgG1 expression vector, and 4G2 and 9E10 in a mouse IgG2 expression vector, were transiently transfected into FreeStyle™ 293F cells and purified as described previously.
Validation	All antibodies were validated by ELISA binding assays to cognate antigens, and virus neutralization assays.

Eukaryotic cell lines

Policy information about cell lines and Sex and Gender in Research

Cell line source(s)	FreeStyle™ 293F cells (ThermoFisher, CAT#R79007) and 293T cells (ATCC, CAT# CRL-3216), derived from a female fetus; Drosophila Schneider S2 cells (ThermoFisher, CAT# R69007); Vero cells (ATCC, CAT#CRL-81), derived from the kidney of a female African green monkey; K562 cells (ATCC, CAT#CCL-243), originally isolated from the bone marrow of a female with chronic myelogenous leukemia.
Authentication	Cells were authenticated by their individual anticipated functionalities.
Mycoplasma contamination	The cell lines were not tested for mycoplasma contaminations.
Commonly misidentified lines (See ICLAC register)	N/A

Animals and other research organisms

Policy information about studies involving animals; ARRIVE guidelines recommended for reporting animal research, and Sex and Gender in Research

Laboratory animals	Mice include C57BL/6 (8- to 9-week-old), BALB/c (6- to 8-week-old), AG129 (6- to 8-week-old), and OmniMouse (20- to 21-week-old)
Wild animals	N/A
Reporting on sex	Since we consider that ZIKV infection can affect pregnancy and cause severe fetus development deficiency, we used female mice for initial study to identify the lead immunogens. Following up studies will include both male and female animals.
Field-collected samples	N/A
Ethics oversight	Animal experiments were carried out in compliance with all relevant U.S. National Institutes of Health regulations and were approved

by the Institutional Animal Care and Use Committees (IACUCs) : at NOBLE LIFE SCIENCES, INC. (IACUC approval number: SVL-197), BIOQUAL (IACUC approval number 20-026), and University of Maryland School of Medicine (IACUC approval number: #0219005).

Note that full information on the approval of the study protocol must also be provided in the manuscript.

Plants

Seed stocks | N/A

Novel plant genotypes | N/A

Authentication | N/A

Flow Cytometry

Plots

Confirm that:

- [x] The axis labels state the marker and fluorochrome used (e.g. CD4-FITC).
- [x] The axis scales are clearly visible. Include numbers along axes only for bottom left plot of group (a 'group' is an analysis of identical markers).
- [x] All plots are contour plots with outliers or pseudocolor plots.
- [x] A numerical value for number of cells or percentage (with statistics) is provided.

Methodology

Sample preparation

Cryopreserved OmniMouse animal splenocytes were quickly thawed in a 37oC water bath, followed by treatment with DNase I (10,000 U/ml, Roche, 1000-fold dilution) in RPMI 1640 with 10% FBS at room temperature for five minutes, and washed with chilled PBS. After staining with Aqua Dead Cell Staining dye (Life Technologies, Cat# L349660, LOT#1941440, 400-fold dilution) in PBS for 15 minutes, followed by two steps of staining procedure, each lasting one hour, cells were washed with PBS between these two staining procedures. Since OmniMouse mice carry a rat Ig constant region encoding sequences adjacent to the human heavy chain variable gene segments, most of the class-switched B cells would bear rat IgG constant region. The first staining medium contains polyclonal goat anti-rat IgG PE/Dazzle 594 conjugate (Biolegend, CAT#405432, LOT#B319517, 100-fold dilution) to identify class-switched B cells.

The second staining medium contained a cocktail of antibodies and antigen for identifying antigen-specific B cells, as described previously with minor modifications 67. Briefly, a cocktail of antibodies diluted in RPMI 1640 /10% FBS, which contains CD3- PerCp/Cy5.5 (clone 17A2, Biolegend, CAT# 100218, LOT#B233420, 500-fold dilution), F4/80-PerCp/Cy5.5 (clone BM8, Biolegend, CAT#123128, LOT#B222447, 1000-fold dilution), Gr1-PerCp/Cy5.5 (clone RB6-8C5, Biolegend, CAT#108428, LOT#B245570, 2000-fold dilution), CD19-APC-Cy7 (clone 1D3, BD Pharmingen, CAT#557655, LOT#6070644, 500-fold dilution), B220- Alexa Fluor® 700 (clone RA3-6B2, BioLegend, CAT#103232, LOT#B242183, 2000-fold dilution), mouse IgG2a-FITC (clone RMG2a-62, Biolegend, CAT#407106, LOT#B199948, 500-fold dilution), mouse IgG2b-FITC (clone R12-3, BD Pharmingen, CAT# 553395, LOT#7096543, 500-fold dilution), mouse IgG1-FITC (clone A85-1, BD Pharmingen, CAT#553443, LOT#7020839, 500-fold dilution), mouse IgD-Pacific Blue (clone RA3-6B2, BioLegend, CAT#405712, LOT#B209676, 1000-fold dilution), and CC_FLE–streptavidin-PE conjugate (at 2.5 ug/ml) was used to stain the cells. CC_FLE-Avi carrying an Avi-tag at the c-terminus were biotinylated with BirA 500 biotin ligase (Avidity, AviTag™ Technology, Aurora, CO), followed by mixing with streptavidin-PE conjugate (Invitrogen, CAT# S21388, LOT#1784902) at 1:1 molar ratio to form CC_FLE–streptavidin-PE conjugate as described previously. As shown in Fig. S10, the stained cells were applied to a BD FACS Aria III cell sorter (BD Biosciences) to sort for antigen-specific class-switched B cells with BD FACSDiva software V8.02 using the phenotype of CD19+/B220+/CD3-Gr1-F4/80-/mouse IgD-IgG-/rat IgG+/CC_FLE+ into 96-well plates at single-cell density, followed by single-cell reverse transcription and PCR reactions to amplify human Ig V(D)J gene segments as previously described

Instrument | BD FACS Aria III cell sorter (BD Biosciences)

Software | FACS data were collected using BD FACSDiva software, and analyzed with FlowJo 10.6.1.

Cell population abundance | Cell population abundance were indicated in the FACS flow chart (Fig. S10). Breifly, 0.26% of memory B cells are antigen-specific.

Gating strategy | Antigen-specific class-switched B cells were gated with the phenotype of CD19+/B220+/CD3-Gr1-F4/80-/mouse IgD-IgG-/rat IgG+/CC_FLE+ (Fig. S10).

- [x] Tick this box to confirm that a figure exemplifying the gating strategy is provided in the Supplementary Information.
